# Prognostic Differences between Men and Women with Acute Coronary Syndrome. Data from a Brazilian Registry

**DOI:** 10.5935/abc.20180166

**Published:** 2018-11

**Authors:** Alexandre de Matos Soeiro, Pedro Gabriel Melo de Barros e Silva, Eduardo Alberto de Castro Roque, Aline Siqueira Bossa, Bruno Biselli, Tatiana de Carvalho Andreucci Torres Leal, Maria Carolina Feres de Almeida Soeiro, Fábio Grunspun Pitta, Carlos V. Serrano Jr., Múcio Tavares Oliveira Jr.

**Affiliations:** 1 Unidade Clínica de Emergência - InCor - HCFMUSP, São Paulo, SP - Brazil; 2 Hospital TotalCor, São Paulo, SP - Brazil; 3 Hospital Metropolitano, Serra, ES - Brazil

**Keywords:** Acute Coronary Syndrome/epidemiology, Prognosis, Gender Indentify, Multicenter Study, Mortality, Hypertension, Percutaneous Coronary Intervention

## Abstract

**Background:**

Gender-related differences have been reported in patients with acute coronary syndrome. The description of this comparative finding in a Brazilian registry has not yet been documented.

**Objective:**

To compare male vs. female patients regarding the baseline characteristics, coronary findings, treatment and in-hospital and long-term prognosis.

**Methods:**

This is a retrospective, multicenter and observational study that included 3,745 patients (2,437 males and 1,308 females) between May 2010 and May 2015. The primary in-hospital outcome was all-cause mortality. The secondary outcome consisted of combined events (cardiogenic shock, reinfarction, death, stroke and bleeding). The comparison between groups was performed using the chi-square and the t test, considering p < 0.05 as significant. In the long term, mortality and combined events were assessed using the Kaplan-Meier method, with a mean follow-up of 8.79 months.

**Results:**

The mean age was 60.3 years for males and 64.6 for females (p < 0.0001). The most prevalent risk factor was systemic arterial hypertension in 72.9% of the women and 67.8% of the men (p = 0.001). Percutaneous coronary intervention was carried out in 44.9% of the males and 35.4% of the females (p < 0.0001), and coronary artery bypass grafting (CABG) was performed in 17% of the males and 11.8% of females (p < 0.0001), with a higher prevalence of three-vessel coronary artery disease in males (27.3% vs. 16.2%, p < 0.0001). Approximately 79.9% of the female patients received a diagnosis of acute coronary syndrome without ST-segment elevation, while in the male patients, this diagnosis was attained in 71.5% (p < 0.0001). No significant differences were observed between the groups in the short and long term, regarding both mortality and the combined events.

**Conclusion:**

Several gender-related differences were observed in patients with acute coronary syndrome regarding the demographic characteristics, coronary artery disease pattern and implemented treatment. However, the prognostic evolution was similar between the groups.

## Introduction

Coronary heart disease and, particularly Acute Coronary Syndrome (ACS), is the leading cause of mortality and morbidity in the Western world, both in women and men. The benefits of early reperfusion therapy for ACS patients are well established. However, recent studies have shown that, according to gender, there may be variations in diagnosis, coronary stratification, and chosen reperfusion method. It has also been shown that women with acute myocardial infarction (AMI) are less likely to undergo reperfusion strategies and clinical treatment than men, and there is a lack of risk awareness among women. Differences in survival between men and women, reported in some studies, may reflect not only the gender bias but also differences in coronary anatomy, age, and comorbidities.^1^^,^^2^

The description of these comparative data between men and women in a Brazilian registry has yet to be documented. This study was developed aiming at comparing ACS male vs. female patients regarding the baseline characteristics, coronary findings, treatment, in-hospital and medium-term prognosis.

## Methods

### Study population

This is a retrospective, multicenter and observational study. A total of 3,745 patients with ACS admitted at an Emergency Sector between May 2010 and May 2015 were included. The patients were divided into two groups: male (n = 2,437) and female gender (n = 1,308). There was no exclusion criterion. All patients were submitted to a coronary angiography within 48 hours of admission.

All patients who met the criteria established by the last Brazilian Society of Cardiology (SBC) and American Heart Association (AHA) guidelines were considered to be SCA patients.^3^^,^^4^ Non-ST elevation ACS (NSTE-ACS) was defined as the presence of chest pain associated with electrocardiographic changes, or rise/fall of troponin at hospitalization, or, in the absence of these, as clinical picture and risk factors compatible with unstable angina (chest pain at rest or at minimal effort, severe or occurring with a crescendo pattern). Major bleeding was defined by types 3 and 5 Bleeding Academic Research Consortium (BARC)^4^ score, and minor bleeding by types 1 and 2. Reinfarction was considered when there was chest pain recurrence associated with a new elevation in troponin levels. Ischemic cerebrovascular accident (iCVA) was considered when the patient had a new focal motor neurological deficit confirmed by cranial computed tomography. The heart failure outcome was considered when hospitalization was associated with the disease or symptoms with functional class ≥ 2, according to the New York Heart Association classification.

The following data were obtained: age, gender, body mass index, presence of diabetes mellitus, systemic arterial hypertension, smoking, dyslipidemia, family history of early coronary disease, heart failure, previous coronary artery disease (AMI, angioplasty or previous CABG), hemoglobin, creatinine, troponin peak, Killip classification, left ventricular ejection fraction, systolic blood pressure, medications used in the first 24 hours of hospitalization and chosen coronary treatment.

All patients were referred to the post-discharge consultation between 14 and 30 days, and to a new consultation in 6 months, undergoing ischemia or catheterization tests, requested according to the medical evaluation of the team in charge. Coronary reintervention was necessary in 7.2% of the male patients and 6.4% of the female patients at the follow-up (p = 0.48). The follow-up was carried out through telephone contact and medical record review. The study was submitted to and approved by the Research Ethics Committee. The Free and Informed Consent form was filled out by all the patients included in the study.

### Statistical analysis

The primary in-hospital outcome was all-cause mortality. The secondary outcome consisted of combined events (cardiogenic shock, reinfarction, death, iCVA and bleeding). A descriptive analysis was performed using means and standard deviations, when using parametric tests, and median and interquartile intervals in non-parametric tests. The comparison between groups was performed using the chi-square test for categorical variables. The unpaired t-test was used for continuous variables, when the Komolgorov-Smirnov normality test showed a normal distribution, considering p < 0.05 as significant. The Mann-Whitney U test was used when the distribution was non-normal. The multivariate analysis was performed by logistic regression only when there was a significant difference between groups in some assessed outcome, considering p < 0.05 as significant. All baseline characteristics shown in Table 1 that showed a significant difference between the groups were considered as variables in the analysis.

**Table 1:** Baseline clinical characteristics of male vs. female patients

Characteristic	Male (n = 2,437)	Female (n = 1,308)	p-value
Age	60.3 ± 11.6	64.7 ± 10.4	< 0.0001^*^
BMI	26.1 ± 6.5	24.3 ± 6.1	< 0.0001^†^
Diabetes Mellitus	1,041 (42.7)	627 (47.9)	0.011^‡^
SAH	1,652 (67,8)	968 (72.9)	0.001^‡^
Smoking	819 (33.6)	332 (25.4)	< 0.0001^‡^
FH positive for CAD	361 (14.8)	171 (12.9)	0.113^‡^
Dyslipidemia	1,136 (46.6)	666 (50.9)	0.011^‡^
Heart failure	214 (8.8)	133 (10)	0.778^‡^
Previous iCVA	124 (5.1)	67 (5.1)	0.925^‡^
Previous AMI	819 (33.6)	378 (28.9)	0.004^‡^
Previous CABG	356 (14.6)	140 (10.7)	0.001^‡^
Previous CA	522 (21.4)	234 (17.9)	0.011^‡^
Hemoglobin, mg/dL	14.6 ± 1.9	13.2 ± 1.7	< 0.001^*^
Peak troponin, ng/dL	11.8 ± 5.9	8.0 ± 7.2	< 0.001^*^
Creatinine, mg/dL	1.3 ± 0.5	1.5 ± 0.4	< 0.0001^*^
SBP, mmHg	134.2 ± 29.4	133.0 ± 27.2	0.104^†^
LVEF,%	52.3 ± 19.9	51.8 ± 18.7	0.09^†^
Killip ≥ 2	212 (8.7)	99 (7.6)	0.259^‡^
ASA	2,383 (97.8)	1,267 (96.9)	0.081^‡^
Beta-blocker	2,149 (88.2)	1,105 (84.5)	0.002^‡^
GPI IIb/IIIa	202 (8.3)	114 (8.7)	0.292^‡^
Enoxaparin	1,859 (76.3)	981 (75)	0.405^‡^
Fondaparinux	258 (10.6)	128 (9.8)	0.46^‡^
Clopidogrel	1,772 (72.7)	920 (70.3)	0.132^‡^
Statins	1,228 (50.4)	647 (49.5)	0.768^‡^
ACE inhibitor	1.694 (69.5)	870 (66.5)	0.065^‡^

Results are expressed as mean ± standard deviation, median ± standard deviation or n (%).

* Unpaired t test;

† Mann-Whitney U test;

‡ chi-square test. BMI: body mass index; SAH: systemic arterial hypertension; FH: family history; CAD: coronary artery disease; iCVA: ischemic cerebrovascular accident; AMI: acute myocardial infarction; CABG: coronary artery bypass grafting; CA: coronary angioplasty; SBP: systolic blood pressure; LVEF: left ventricular ejection fraction; ASA: acetylsalicylic acid; GPI: glycoprotein inhibitor; ACE inhibitor: angiotensin-converting enzyme inhibitor.

The medium-term analysis was performed by Log-rank using Kaplan-Meier curves to assess the difference between the groups, with a mean follow-up of 8.79 months. A total of 274 patients were lost to follow-up. The evaluated outcomes were combined events (reinfarction, death and heart failure). A p value < 0.05 was considered significant. The multivariate adjustment was performed only when there was a significant difference between groups in some evaluated outcome.

All calculations were performed using the Statistical Package for Social Science (SPSS), version 10.0.

## Results

The mean age was 60.3 years for males and 64.6 for females (p < 0.0001). The most prevalent risk factor was systemic arterial hypertension, observed in 72.9% of the women and 67.8% of the men (p = 0.001). The baseline characteristics of the study population are shown in table 1.

Regarding the treatment, percutaneous coronary intervention was performed in 44.9% of the males and 35.4% of female patients (p < 0.0001). Coronary artery bypass grafting was performed in 17.0% of the men vs. 11.8% of the women (p < 0.0001). Regarding the coronary artery disease pattern and the clinical presentation, significant differences were observed between the male and female groups, with 27.3% vs. 16.2% with a three-vessel pattern (p < 0.0001), 18.9% vs. 19.9% with a two-vessel pattern (p = 0.381), 28.5% vs. 20.1% of STE-ACS (p = 0.01) and 71.5% vs. 79.9% of non-ST elevation ACS (NSTE-ACS), respectively (p < 0.0001).

Regarding the comparison of in-hospital outcomes, there were no significant differences between the groups regarding mortality (3.1% vs. 3.7%, p = 0.293) and the combined events (12.2% vs. 12, 0%, p = 0.885), respectively, between males and females (Table 2).

**Table 2:** Univariate analysis comparing different in-hospital outcomes between male vs. female patients

Outcomes	Male (n = 2,437) n (%)	Female (n = 1,308) n (%)	p-value
Reinfarction	24 (1.0)	14 (1.1)	0.519
Cardiogenic shock	107 (4.4)	41 (3.1)	0.066
Bleeding	73 (3.0)	47 (3.6)	0.655
iCVA	17 (0.7)	7 (0.5)	0.678
Mortality	76 (3.1)	48 (3.7)	0.293
Combined events	297 (12.2)	157 (12.0)	0.885

iCVA: ischemic cerebrovascular accident.

**Figure 1:**
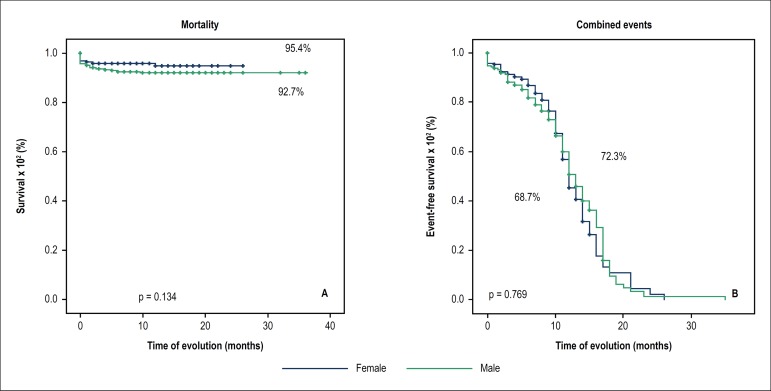
Event-free survival and percentage of combined events in the medium-term comparison between males and females.

The medium-term follow-up did not show a significant difference regarding combined events in the male and female groups (31.3% vs. 27.7%, p = 0.769), or in relation to mortality, respectively (Figure 1 and Table 3).

**Table 3:** Comparison of different medium-term outcomes between the groups of male vs. female patients

Outcomes	Male (n = 2,256) n (%)	Female (n = 1,215) n (%)	p-value
Reinfarction	183 (8.1)	77 (6.3)	0.980
Heart Failure	359 (15.9)	204 (16.8)	0.783
Mortality	165 (7.3)	56 (4.6)	0.134
Combined events	706 (31.3)	337 (27.7)	0.769

## Discussion

The study showed important data found in the Brazilian population, which are consistent with the results of recent publications in the literature. Significant differences were observed regarding the presence of a greater number of risk factors and older age in the female group. Higher rates of reperfusion (percutaneous or surgical) and ST-elevation ACS in men in comparison to women have also been reported as being significant. Regarding mortality and combined events, there were no significant differences between male and female patients in the short and medium-term.

It is estimated that 43 million women have coronary artery disease, which is the leading cause of death in women, with approximately 400,000 deaths per year in the United States.^5^ Nearly 43% of ACS patients are women, with approximately 360,000 women submitted to Percutaneous Coronary Intervention (PCI) only in 2007.^5^ The number of women with ACS (34.9%) found in this study is proportionally lower than the data published in most international studies. One of the hypotheses for this fact is that there is still a reasonable index of diagnostic error regarding ACS in women, perhaps more pronounced in Brazil, due to the difficulty of access to health care services. Some studies make it clear that the clinical manifestations of coronary disease in women are sometimes non-specific and/or underrated, and a large number of female patients are discharged without a correct diagnosis.^2^

Another interesting finding of this study was the fact that the group of women, in addition to being older, also had a higher number of comorbidities, such as diabetes mellitus, hypertension and dyslipidemia. Women, in most instances, are older when they exhibit their first manifestation of ACS, at a mean age of 71.8 years, compared to 65 years for men.^2^^,^^5^^-^^10^ The older age of onset in women, when compared to men, is probably due to the protective role of estrogen circulation in the vascular endothelium. This hypothesis derives mainly from the observation that the incidence of AMI increases substantially in postmenopausal women. The effects of estrogen on the vascular system include increased nitric oxide release, which leads to vasodilation, prostaglandin production regulation, and smooth muscle proliferation inhibition.^2^ Corroborating these data, a retrospective study in patients with STE-ACS showed that women were significantly older (70.9 years vs. 63 years, p < 0.001) and more often had diabetes mellitus (36.2% vs. 21.0%, p < 0.001) and hypertension (82.3% vs. 73.7%, p = 0.006).^6^

As for the ACS presentation, due perhaps to the greater number of comorbidities and the older age at presentation, women classically had a higher proportion of NSTE-ACS when compared to men.^2^^,^^5^^,^^7^^-^^9^^,^^11^^,^^12^ In a retrospective cohort published in 2015, Worrall-Carter et al.,^8^^)^ assessed 28,985 patients with ACS, showing that the diagnosis of NSTE-ACS was more prevalent among women than men (86% vs. 80%; p < 0.001).^8^ In another study, with 7,304 patients, the higher prevalence of NSTE-ACS in women was repeated, accounting for 70.7% of the presentations in the female gender (p < 0.01).^9^ As observed in our study, the findings in the Brazilian population follow the same global trends regarding the clinical/ electrocardiographic presentation of ACS between the genders.

The coronary anatomy in female patients tends to be less complex, with a lower prevalence of three-vessel disease described in female patients, similarly to our results. The description of the three-vessel coronary artery pattern varies from 15.4% to 36.8% in females, and from 20.5% to 40.8% in males, always with a significant difference in the different analyses.^9^^,^^13^^,^^14^ However, despite the theoretic simpler anatomy regarding the percutaneous coronary reperfusion approach, women are less frequently referred for appropriate treatment in comparison to men.

Regardless the treatment strategy, either with thrombolytic therapy or PCI, women generally have worse outcomes than men. These data become controversial, as women have a more favorable outcome with PCI compared to thrombolytic therapy in the STE-ACS scenario and clearly benefit from an early invasive strategy in any situation.^1^^,^^8^^,^^12^^,^^14^ As an example, a registry published in 2007 on patients with ACS showed that women underwent PCI less frequently than men (Odds Ratio - OR = 0.65; 95% Confidence Interval - 95%CI: 0,61-0,69), and their in-hospital mortality showed a worse index (10.7% vs. 6.3%, p < 0.001).^1^ This description in the literature is once again reinforced by the data from our study, showing higher rates of surgical and percutaneous revascularization in men. The most plausible explanation for this scenario is that women are more likely to have unusual pathophysiological mechanisms of coronary disease, such as spontaneous coronary artery dissection or coronary artery spasm. Furthermore, the fact that they have more comorbidities, such as diabetes and dyslipidemia, favors the occurrence of lesions in thinner vessels and more extensive lesions.^2^

Finally, in the present study, we did not find any prognostic differences, either in-hospital or in the medium term, between the genders in our population. Some studies follow the same line and also have not shown any significant differences between the genders regarding mortality in ACS.^6^^,^^8^^,^^9^^,^^11^^,^^13^^)^ Reinforcing our finding, a study published in 2012 with 1,640 patients with ACS showed no differences in cardiovascular mortality according to gender (1.3% vs. 2.7%, p = 0.18) at the end of one year after PCI for men and women, respectively.^13^ Finding similar mortality rates between men and women in a context of less invasive treatment in the female group may seem odd. However, drug treatment adequacy, early diagnosis and distinct pathophysiology between the genders may help to explain this finding.^14^

Nevertheless, in most studies, regardless of age, within 1 year after the first AMI, more women died when compared to men (26% vs. 19%), with similar results after 5 years (47% vs. 36%).^2^^,^^5^^,^^7^^,^^15^ In one of the largest registries ever published on the subject, more than 2 million patients submitted to CABG were analyzed, comparing the prognosis between the genders. Unadjusted in-hospital mortality was higher in women (3.2% vs. 1.8%, p < 0.001). The female gender remained an independent predictor of mortality after the multivariate adjustment (OR = 1.40, 95%CI: 1.36-1.43, p < 0.001) in all age groups. However, an interesting result was the observation that in-hospital mortality declined at a faster rate in women (3.8% to 2.7%) than in men (2.2% to 1.6%) between 2003 and 2012.^15^

### Limitations

Despite the large sample, this study is retrospective and has a much higher number of male patients in relation to the female group. Such differences are based on the actual incidence of ACS in the population and also on the failure to recognize the disease in women. Also, we do not have a description of the type of vascular access used, something that may influence the rate of bleeding associated with the percutaneous coronary intervention. Unusual manifestations of coronary disease, such as spasm or spontaneous dissection, were not described separately. The loss to follow-up of 7.3% of the patients may have influenced the results. Finally, patients with systemic diseases or neoplasias were not excluded, which could have influenced survival.

## Conclusion

Multiple gender-related differences were observed in patients with acute coronary syndrome, regarding demographic characteristics, coronary artery disease pattern and implemented treatment. However, the in-hospital and medium-term prognostic evolution was similar between the groups.
